# *Pneumocystis jirovecii* pneumonia complicated a case of SARS‐CoV‐2 infection and multiple sclerosis after treatment with rituximab

**DOI:** 10.1002/ccr3.7455

**Published:** 2023-06-02

**Authors:** Mahmoud Sadeghi Haddad Zavareh, Hamed Mehdinezhad, Rahele Mehraeen, Mohammad Golparvar Azizi, Ali Tavakoli Pirzaman

**Affiliations:** ^1^ Infectious Diseases and Tropical Medicine Research Center, Health Research Institute Babol University of Medical Sciences Babol Iran; ^2^ Department of Internal Medicine, Rouhani Hospital Babol University of Medical Sciences Babol Iran; ^3^ Department of Radiology, Rohani Hospital Babol University of Medical Sciences Babol Iran; ^4^ Student Research Committee Babol University of Medical Sciences Babol Iran

**Keywords:** rituximab, COVID‐19, multiple sclerosis, *Pneumocystis jirovecii* pneumonia, SARS‐CoV‐2 infection

## Abstract

Although immunodeficient patients are less prone to develop Coronavirus disease 2019 (COVID‐19)‐mediated cytokine storm, secondary infections can cause serious complications in this vulnerable population. They are more likely to develop opportunistic infections that can mimic the symptoms of the severe acute respiratory syndrome coronavirus 2 (SARS‐CoV‐2) infection. Herein, we presented a 27‐year‐old male patient of SARS‐CoV‐2 infection, who was complicated with *Pneumocystis jirovecii* pneumonia (PJP), following treatment with rituximab. First, he was hospitalized for 5 days with fever, cough, and dyspnea due to COVID‐19 infection, and treated with remdesivir and glucocorticoid. Then, he has been referred to our center with cough, dyspnea, body pain, and fever. Due to persistent fever, the progression of pulmonary lesions, and reduced oxygen saturation, we began treatment with piperacillin + tazobactam, vancomycin, and levofloxacin. Nevertheless, the patient's fever did not stop after the aforementioned empiric treatment and his condition got worse and he was admitted to the intensive care unit. The result of BAL fluid, tested for *P. jirovecii* by RT‐PCR, turned out to be positive. Therefore, we started trimethoprim‐sulfamethoxazole and dexamethasone, which improved his condition. We hope this article helps clinicians consider causes other than COVID‐19, especially opportunistic infections such as PJP, in patients with respiratory symptoms and fever.

## 1 INTRODUCTION

In December 2019, the viral infection outbreak in Wuhan, China became a global pandemic that crossed borders and put the lives of populations in danger, especially those with immunodeficiency.^1^ During these challenging years and the management of COVID‐19 disease, many questions have arisen regarding immunomodulatory and immunosuppressive therapies, especially, in patients with demyelinating disease.^2^ Given the reduced immune responses, immunodeficient patients are less prone to develop COVID‐19‐mediated cytokine storms. However, this vulnerable population can suffer serious complications caused by secondary infections. Plus, they are more likely to develop opportunistic infections that can mimic the symptoms of SARS‐CoV‐2 infection.^3^

Multiple sclerosis (MS), an autoimmune, demyelinating disease of the central nervous system (CNS), is mainly linked to the reaction of CD4+ T cells to the antigens of the myelin membrane.^4^ Two previous randomized clinical trials (RCTs) have demonstrated that rituximab, a monoclonal anti‐CD20 antibody, could be effective in primary progressive multiple sclerosis (PPMS) and relapsing–remitting multiple sclerosis (RRMS).^5^ However, there are growing reports, introducing post‐rituximab therapy *Pneumocystis jirovecii* pneumonia (PJP), in cases of systemic lupus erythematosus, Wegener's granulomatosis, rheumatoid arthritis, lymphoma, and humoral renal transplant rejection.^6^ Given the fact that rituximab is a commonly used medication for the highly active forms of MS, it is important to consider the possible risk of PJP in these patients.

In the current study, we presented a case of COVID‐19 infection, that was complicated with PJP, following treatment with rituximab for RRMS.

## 2 CASE PRESENTATION

A 27‐year‐old male patient with MS for the past 12 years has been referred to our center with complaints of cough, dyspnea, myalgia, epigastric pain, nausea and vomiting, and fever, in April 2022. In June 2021, he was also hospitalized in another center for 5 days with fever, cough, and dyspnea due to COVID‐19 infection, and treated with remdesivir and glucocorticoid. Eventually, he was discharged, 24 hours after the termination of his fever.

He had also a previously‐diagnosed RRMS for the past 12 years, which resulted in neurological symptoms such as blurred vision, headaches, and paresthesia in the lower limbs. Due to the uncontrollable MS, intravenous (IV) injections of rituximab (1 gr per 6 month) was applied. The last injection was in February 2022. He was also taking prednisolone (5 mg daily) for maintenance therapy and other medications such as salmeterol plus fluticasone inhaler, montelukast, NAC, and aspirin.

On physical examination, his temperature was 38°C; blood pressure, 125/80 mmHg; respiratory rate, 17 breaths per min; pulse rate, 88 beats per min; and oxygen saturation, was 94% (without any oxygen therapy). His fever was during the day, but the sweating was preferably overnight. He also had dyspnea that made him unable to walk (FCIII). The patient's cough contained yellow sputum, and the body pain was responsive to analgesic. He had symmetric chest wall movements and no crackles or wheezing was detected. However, we could find rhonchi. Despite there being no evidence of organomegaly, epigastric tenderness was detected in the examinations of the abdomen. In the neurological examinations, no abnormal findings were detected. On admission, his laboratory data showed an increased LDH (2061 U/L) along with increased levels of hepatic biomarkers (Table 1). Moreover, the levels of CRP, ESR, and PCT were higher than the normal range. Transthoracic echocardiography (TTE) revealed an increased pulmonary artery pressure (30 mmHg). PCR test for COVID‐19 was negative on admission. First, considering the patient's fever and pneumonic pattern along with the air bronchogram, we strongly suspected a secondary bacterial infection. Considering that the most common secondary infection is pneumococcal, empiric treatment with ceftriaxone was started to cover this infection. Also, because he was hospitalized for less than a week in the previous hospital, the possibility of nosocomial infection was not given. However, due to the persistent fever, progression of radiological lesions, reduced oxygen saturation during empiric treatment with ceftriaxone, and the possibility of hospital‐acquired pneumonia (HAP), the combination of piperacillin + tazobactam and levofloxacin was chosen in order to cover resistant gram‐negative bacilli. Vancomycin was also started for the patient to cover resistant *Streptococcus pneumoniae* and *Staphylococcus aureus* infections. Despite receiving the previous antibiotic regimen, the patient's fever did not stop and the lung lesions were still progressing, so changing the treatment regimen to a drug combination with a wider coverage was considered, and also, according to the sensitivity pattern of gram‐negative organisms of this center, the combination of imipenem and linezolid was prescribed. Especially since there was a higher risk of *Acinetobacter* in our center.

**TABLE 1:** Laboratory data on admission and discharge days.

	Admission day	Discharge day
WBC (per μL)	4600	7000
Neutrophil (%)	89	68
Lymphocyte (%)	8	25
Hb (mg/dL)	13.2	11.5
PLT (per μL)	256,000	277,000
LDH (U/L)	2061	826
CRP (mg/dL)	95	14
ESR (mm/h)	26	28
Procalcitonin (ng/mL)	0.21	0.16
Bilirubin (total) (mg/dL)	1	0.6
Bilirubin (direct) (mg/dL)	0.4	0.2
AST (U/L)	96	39
ALT (U/L)	132	84
ALP (U/L)	291	272
Troponin	Negative	–
BUN (mg/dL)	13	14
Creatinine (mg/dL)	1.2	0.9
PT (sec)	14.2	12
PTT (sec)	40	30
INR	1.3	1

Abbreviations: ALP, alkaline phosphatase; ALT, alanine transaminase; AST, aspartate transaminase; BUN, blood urea nitrogen; CRP, C‐reactive protein; ESR, erythrocyte sedimentation rate; Hb, hemoglobin; INR, international normalized ratio; LDH: lactate dehydrogenase; PLT, platelets; PT, prothrombin time; PTT, partial thromboplastin time; WBC, white blood cells.

**FIGURE 1:**
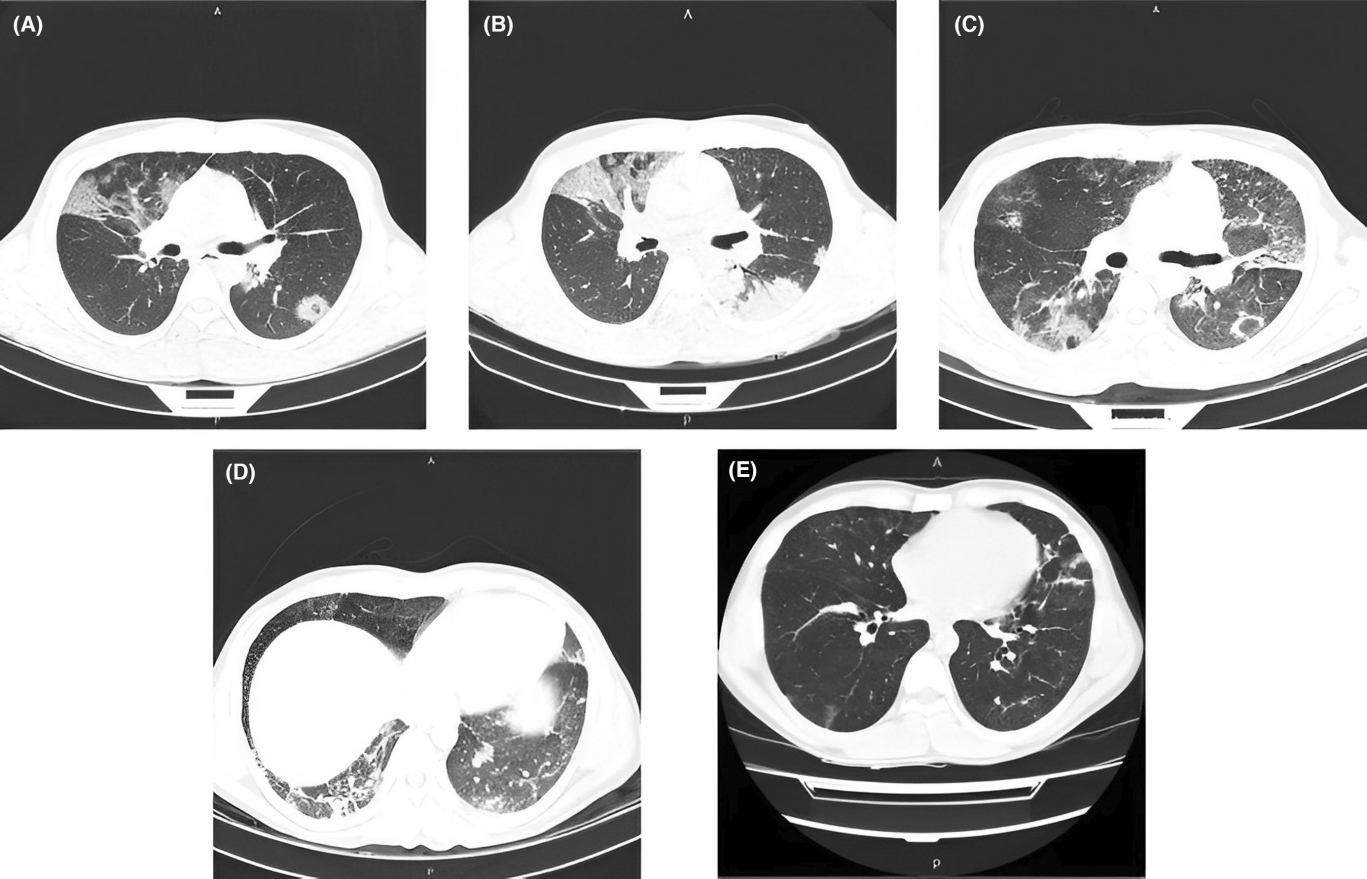
(A) on admission day CT scan, there were multilobal bilateral patchy consolidations, ground glass opacities, and interlobular septal thickening, with the peripheral and peri‐bronchovascular distribution. “Crazy paving” is the dominant feature at the right upper lobe; (B) CT scan right before bronchoscopy showed increased consolidations, ground glass opacities, and crazy paving in the same manner at multiple lung segments; (C, D) on discharge day CT scan, there were decreased consolidations and change to ground glass opacities and crazy paving appearance at the same involved segments. There was also evidence of “reverse halo” in several segments and progression to fibrotic changes predominantly at lung bases, as parenchymal and subpleural bands; (E) CT scan image, 3 months after discharge.

We were highly suspicious of opportunistic infections such as Cytomegalovirus, tuberculosis (TB), pulmonary aspergillosis, and *P. jirovecii*. We also considered the probability of infection in other sites. Thus, echocardiography and abdominal and pelvic ultrasonography were performed and revealed to be normal. Accordingly, bronchoalveolar lavage (BAL) fluid was obtained through bronchoscopy and was assessed for infections. In the case of TB, both the BAL smear and PCR were negative. Aspergillosis was also excluded due to a negative level of galactomannan in the BAL. PCR test for COVID‐19 was negative, too. Further, the cytological assessment of BAL fluid showed no evidence of malignancy. Pneumomediastinum and pneumothorax were also excluded from the CT scan images. In Figure 1, the patient's CT scan findings were demonstrated, on admission day, right before bronchoscopy, discharge day, and 3 months later.

On the 12th day, the patient was transferred to the intensive care unit and was given oxygenation using a reservoir bag and high‐flow nasal cannula (HFNC) because of the decline in the oxygen saturation (SpO2 = 88%) and being put in the critically ill group. Moreover, the arterial blood gas (ABG) test showed compensated respiratory alkalosis despite the treatment measures. At this time, the result of BAL fluid, tested for *P. jirovecii* by RT‐PCR, turned out to be positive. Therefore, we started trimethoprim–sulfamethoxazole (the treatment of choice for PCP) and dexamethasone. Following the treatment, his condition improved and the levels of PCT and CRP decreased. He was discharged in May 2022.

## 3 DISCUSSION

Respiratory viral infections can put patients at the risk of secondary infections, especially by bacterial and fungal organisms.^7^ In a previous study, about 30% of SARS‐CoV‐2 infected cases were at risk of developing secondary pneumonia without a known reason.^8^ In fact, SARS‐CoV‐2 infection can interfere with the immune system and its balance; therefore, it may result in an increased risk of fungal infections such as invasive candidiasis, pulmonary aspergillosis, and *P. jirovecii*.^9^ Given the fact that Pneumocystis pneumonia (PCP) and COVID‐19 may have similar and common clinical features such as profound hypoxemia and bilateral multifocal infiltrates, coinfection with PJP could be missed, especially in those with life‐threatening forms of COVID‐19 infection. Hence, it seems wise to apply additional diagnostic workup for PJP in severe COVID‐19 patients, especially in the presence of clinical features that support coinfection, like cystic formations on chest CT scan and an increased level of lactate dehydrogenase, even if there were no risk factors for PJP.^10^

It seems that immunosuppression plays an important role in the association of COVID‐19 and PCP. Although impaired immune balance may be useful in the context of COVID‐19 severity, due to the reduced immune response and inflammation, which are related to the severity of manifestations, it is also a chief risk factor for the occurrence of PCP.^11^ In that case, preexisting immunodeficiency (e.g., HIV‐ or drug‐induced) could increase the risk of COVID‐19 and PJP coinfection. Importantly, it might be observed in those who are not included in the known risk groups, which could be a result of severe COVID‐19‐induced lymphopenia or immunosuppressive therapy.^12^

PJP has two morphological forms in its life cycle, including cystic and trophic forms. It is an infection usually identified in patients with impaired T‐cell immunity, particularly CD4^+^ lymphopenia. Unfortunately, severe COVID‐19 infection is associated with severely diminished levels of CD4^+^ cells,^9^ which makes these patients highly susceptible to PJP. Moreover, COVID‐19 infection could result in conditions like acute respiratory distress syndrome, which requires immunosuppressive therapies (e.g. corticosteroids), a familiar risk factor for developing PCP.^9^

## 4 CONCLUSION

SARS‐CoV‐2 infection can interfere with the immune system and its balance; therefore, it may result in an increased risk of fungal infections such as invasive candidiasis, pulmonary aspergillosis, and PJP. In the current study, we presented a case of COVID‐19 infection, that was complicated with PJP, following treatment with rituximab for RRMS. We hope this article helps clinicians consider causes other than COVID‐19, especially opportunistic infections such as PJP, in patients with respiratory symptoms and fever.

## AUTHOR CONTRIBUTIONS

**Mahmoud Sadeghi Haddad Zavareh:** Data curation; supervision; writing – review and editing. **Hamed Mehdinezhad:** Data curation; supervision; writing – review and editing. **Rahele Mehraeen:** Supervision; writing – review and editing. **Mohammad Golparvar Azizi:** Writing – original draft. **Ali Tavakoli Pirzaman:** Data curation; supervision; writing – original draft; writing – review and editing.

## FUNDING INFORMATION

This study did not receive any specific grant from funding agencies in the public, commercial, or not‐for‐profit sectors.

## CONFLICT OF INTEREST STATEMENT

The authors declare no conflict of interest.

## ETHICAL APPROVAL

For the publication of this article, we obtained written informed consent from the patient to release any potentially identifiable data.

## Data Availability

The Data supporting the findings of this study are available upon request from the corresponding author and with permission from Babol University of Medical Sciences, Babol, Iran.
